# Chemosensory Functions in Patients with Inflammatory Bowel Disease and Their Association with Clinical Disease Activity

**DOI:** 10.3390/nu14173543

**Published:** 2022-08-27

**Authors:** Xingyu Han, Ayda-Ayleen Ordouie, Renate Schmelz, Thomas Hummel

**Affiliations:** 1 Smell and Taste Clinic, Department of Otorhinolaryngology, Technische Universität Dresden, Fetscherstrasse 74, 01307 Dresden, Germany; 2 Department of Otolaryngology-Head and Neck Surgery, Beijing Anzhen Hospital, Capital Medical University, No. 2 Anzhen Road, Beijing 100029, China; 3 Medical Department I, Gastroenterology, University Hospital, Technische Universität Dresden, Fetscherstrasse 74, 01307 Dresden, Germany

**Keywords:** olfactory and gustatory function, smell disorder, taste disorder, inflammatory bowel disease, autoimmune diseases

## Abstract

Purpose: Decreased olfactory and gustatory functions are present in various systemic autoimmune diseases. However, little is known about the chemosensory functions of patients with inflammatory bowel disease (IBD). The present study aimed to investigate olfactory and gustatory functions in patients with IBD and their correlation with clinical disease activity. Methods: A total of 103 patients with IBD were included (52 men, 51 women, mean age 40.3 ± 1.2 years) in the present study. Chemosensory functions were assessed utilizing the “Sniffin’ Sticks” olfactory function test and “taste sprays” gustatory function test. The clinical disease activity of patients was graded as remission, mild, and moderate–severe. In addition, inflammatory markers (fecal calprotectin, C-reactive protein and blood leucocyte count) were recorded. Results: In total, 70% of IBD patients were normosmic, 30% were hyposmic, and none of them was functionally anosmic; 6% of the patients showed signs of hypogeusia. Patients with moderate–severe IBD reached a higher olfactory threshold score compared with patients with remission (*p* = 0.011) and mild IBD (*p* < 0.001). The BMI of IBD patients was inversely correlated with their olfactory threshold (r = −0.25, *p* = 0.010). Olfactory and gustatory function in IBD patients did not correlate with duration of disease, blood leucocyte count, CRP level, or fecal calprotectin level. However, patients’ olfactory function significantly increased after 4 months of TNF-α inhibitor treatment (*p* = 0.038). Conclusions: IBD patients are more likely to present with hyposmia. Olfactory thresholds were mainly affected. They were significantly associated with clinical disease activity and BMI. As shown in a subgroup, treatment with TNF-α inhibitors appeared to improve olfactory function.

## 1. Introduction

Inflammatory bowel disease (IBD) is an autoimmune disease, comprising ulcerative colitis (UC) and Crohn’s disease (CD), characterized by chronic relapsing gastrointestinal inflammation whose pathogenesis is not fully understood. It affects approximately 7 million people worldwide with the incidence rising all over the world in industrialized countries [1]. Genetics, environmental exposures, microbiota dysbiosis, and immunity dysregulation contribute to IBD pathogenesis [2]. IBD is a systemic disease, with diverse extra-intestinal manifestations besides gastrointestinal symptoms, including arthritis, uveitis, and skin lesions [3,4]. Associated diseases such as psoriasis, chronic obstructive pulmonary disease, and multiple sclerosis show similarities in pathophysiology [5,6,7].

Previous studies showed that patients with autoimmune diseases have higher rates of chemosensory dysfunction compared to the general population (i.e., type 1 diabetes, rheumatoid arthritis, primary Sjogren’s syndrome, and spondyloarthritis) [8,9,10,11,12]. Neuroinflammation in the olfactory and gustatory systems plays an important role in the pathogenesis of smell and taste disorders by inducing neuron apoptosis and inhibiting neuroregeneration in patients with autoimmune diseases [13,14].

Furthermore, olfactory and gustatory disorders are associated with dietary changes and eating disorders [15,16,17,18]. In fact, patients with hyposmia (decreased olfactory function) and hypogeusia (impaired gustatory function) tend to take in high amounts of saturated fats and carbohydrates as well as fewer vegetables [19,20]. Dietary changes may play a part in the development and progression of IBD with high-energy diets possibly modifying the intestinal microbiota and activating the inflammation of the intestine [21].

Hence, there seems to be a connection between systemic inflammation, dietary parameters and chemosensory functions. Still, little is known about the status of chemical senses in IBD patients. Therefore, the purpose of this study was to explore olfactory and gustatory function in patients with IBD and their correlation with clinical disease activity.

## 2. Methods

This study was performed at the Interdisciplinary Smell and Taste Clinic of the Department of Otorhinolaryngology and the Division of Gastroenterology, Department of Medicine I at the Dresden Technical University Hospital. Patients were recruited at these sites. The study design was approved by the Ethics Committee at the University Clinic of the TU Dresden (application number EK 502112015).

In total, 103 patients with IBD (52 men, 51 women, mean age 40.3 ± 1.2 years) were included in the present study after giving written informed consent. The diagnosis of the patients with UC or CD was made according to ECCO-ESGAR Guideline for Diagnostic Assessment in IBD [22]. The clinical disease activity of patients was graded as remission, mild, and moderate–severe according to clinical scores (partial Mayo score for UC and Harvey Bradshaw Index for CD, respectively) and fecal calprotectin level [23,24]. In CD, a Harvey Bradshaw Index of five to seven indicates mild activity and a score of more than seven points constitutes moderate to severe inflammation. In UC, a partial Mayo score of one to four points displays mild disease activity and five to nine points for a moderate to severe flare. Only patients without acute or chronic nasal disease or dementia were admitted.

Information about the duration of the disease and medication use was collected and analyzed. A standardized depression scale was used to obtain symptoms of depression (Allgemeine Depressionsskala—ADS-L [25], a revised German translation of the Center for Epidemiological Studies Depression Scale—CES-D [26]). Markers of inflammatory activity (fecal calprotectin, C-reactive protein and blood leucocyte count) of the patients were recorded.

Smell function was assessed by the validated “Sniffin’ Sticks” olfactory test (see below). The participants were asked to screen their gustatory function with “taste sprays”. The subjects were required not to eat or drink anything except water and not to smoke within 30 min before the start of the testing to avoid chemosensory desensitization. A small subgroup of 12 patients was examined twice, before and after 4 months of treatment with TNF alpha antagonists.

### 2.1. Olfactory Function Assessment

To measure the patients’ olfactory function, the standard “Sniffin’ Sticks” test kit (Burghart GmbH, Holms, Germany) was utilized [27]. The “Sniffin’ Sticks” test consisted of three different subtests for odor threshold (OT), odor discrimination (OD), and odor identification (OI), which were based on a presentation of the felt-tip pens containing odor. The felt-tip pens were placed about 2 cm in front of the subject’s anterior nostrils for 3 s.

Testing began with the threshold subtest, consisting of pens with 16 different concentrations of phenyl ethyl alcohol (PEA). The participant was asked to choose the pen containing odor from given three pens in a three-alternative forced choice (3-AFC) task. For measuring OT, a single-staircase technique was used [28]. Then, the OD test, in which subjects were required to identify the pen that smelled different from the three pens (3-AFC), was conducted. Lastly, the OI test with 16 familiar odors was performed. The participants needed to identify the odor of the given pen based on the list of four verbal descriptors. The sum of the scores of the four sub-tests made up composite threshold, discrimination and identification (TDI) scores. The TDI scores ranged from 1 to 48 points. Functional anosmia (absence of all olfactory function) was diagnosed if the TDI scores were ≤16. A TDI score between 16.25 and 30.5 was regarded as hyposmia (reduced olfactory function) and above 30.5 as normosmia (normal olfactory function) [29].

### 2.2. Gustatory Function Screening

Screening of gustatory function was conducted utilizing “taste sprays”, a supra-threshold gustatory test. The taste sprays were composed of four natural tastants, including sour: citric acid (0.5 g in 10 g water); sweet: sucrose (1 g in 10 g water); bitter: quinine hydrochloride (0.005 g in 10 g water); and salty: sodium chloride (0.75 g in 10 g water). Umami was not included in the routine taste screening test since many people are not familiar with it [30]. Each tastant was sprayed onto the middle of the tongue of the subjects (about 150 µL per spray) and the subjects were required to identify the taste of sprays as one of the four basic taste qualities [31,32]. Every spray was applied only once. After each spray, subjects were required to rinse their mouth with pure water. The sum of the correctly identified taste number was regarded as the taste scores (ranging from zero to four). The presence of hypogeusia (decreased gustatory function) was assumed if the scores were <4 [28].

### 2.3. Statistical Analysis

Data were analyzed utilizing SPSS 28.0 (SPSS Inc., Chicago, IL, USA). *p* < 0.05 was regarded as a statistically significant difference. An analysis of variance (ANOVA) was conducted to explore differences between patients with remission, mild, and moderate–severe IBD. Paired t-tests were performed to investigate chemosensory function change after 4 months of TNF-α inhibitor treatment. Pearson correlation analysis was used to explore the possible relation between chemosensory function, age, BMI, disease duration, depression severity, and clinical biochemical indexes. Stepwise multiple linear regression analysis was employed to assess the association between olfactory threshold scores and the possible modulators, including age, gender, BMI, clinical disease activity grade, duration of disease, smoking status, medication use, and relevant clinical biochemical indexes.

## 3. Result

### 3.1. Demographics and Clinical Characteristics of Patients with Inflammatory Bowel Disease

Demographics and clinical characteristics of patients are summarized in Table 1; 103 patients with inflammatory bowel disease (IBD) were investigated. A total of 41 (39.8%) patients had UC and 62 (60.2%) patients had CD. Of the patients with IBD, clinical disease activity was graded as remission in 38 (36.9%) patients, mild in 45 (43.7%) patients, and moderate to severe in 20 (19.4%) patients. There was no difference in age, gender distribution, and BMI between the groups. The duration of disease in the mild group (*p* = 0.014) and the moderate–severe group (*p* = 0.001) were both significantly lower than in the remission group. The ADS-L depression scores in the moderate–severe group were significantly higher than in the remission (*p* = 0.016) and mild groups (*p* = 0.048). In addition, there was a marginally significant difference in blood Leucocytes count (*p* = 0.06) and fecal Calprotectin (*p* = 0.06) between the three groups.

### 3.2. Olfactory Function and Gustatory Function of IBD Patients

A total of 72 (70%) were normosmic, 31 of the studied IBD patients (30%) were hyposmic, and none of them was functionally anosmic (Table 2). Compared to normative data for different age groups [29], the patients with IBD exhibited a decreased olfactory function in general. In total, six (6%) of the studied patients showed signs of hypogeusia.

**Figure 1:**
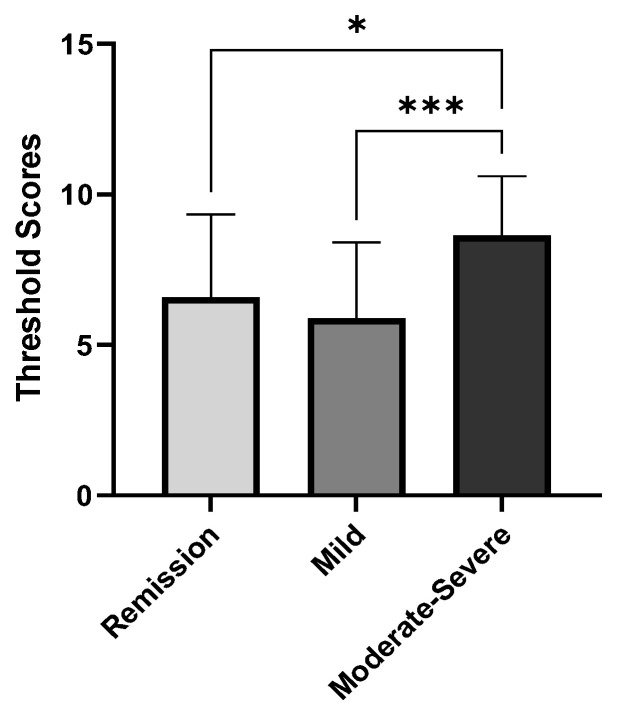
Patients with moderate–severe IBD reached higher olfactory threshold scores compared to patients with remission and mild IBD. * *p* < 0.05; *** *p* < 0.001.

Chemosensory function differences between patients with remission, mild, and moderate–severe IBD are shown in Table 3. Patients with moderate–severe IBD reached a significantly higher olfactory threshold score compared with patients with remission (*p* = 0.011) and mild IBD (*p* < 0.001) (shown in Figure 1). In addition, the TDI scores of patients with moderate–severe IBD also tended to be higher than scores from patients with mild IBD (*p* = 0.052). The olfactory discrimination scores, olfactory identification scores, and taste spray scores were not statistically different between the groups.

### 3.3. Associations between Chemosensory Function and Clinical and Laboratory Indexes in IBD Patients

**Figure 2:**
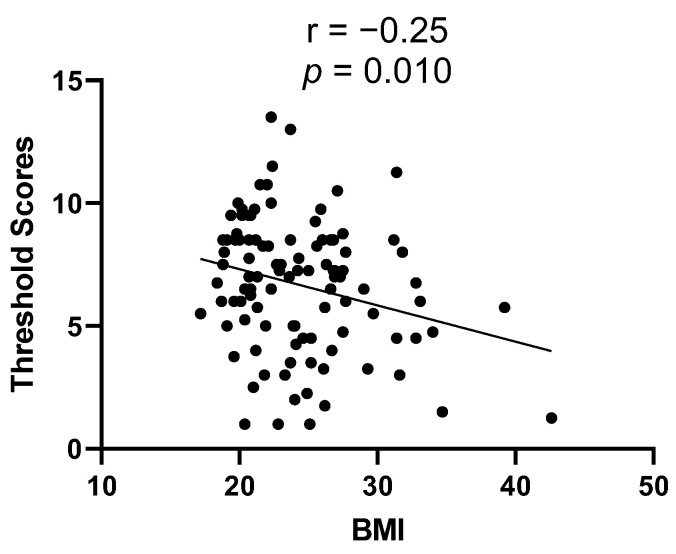
The correlation between olfactory threshold scores and BMI.

The associations between the scores of chemosensory functions and the clinical and laboratory variables are shown in Table 4. The BMI of IBD patients was inversely correlated with their olfactory threshold (r = −0.25, *p* = 0.010, Figure 2). The olfactory and gustatory function in IBD patients did not correlate with the duration of disease, blood leucocyte count, CRP level, or fecal calprotectin level. A best-fit model constructed by multiple regression analysis (Table 5) found that the BMI and clinical disease activity grade were independent predictors of olfactory threshold scores of IBD patients (*p* = 0.006, *p* = 0.018, respectively). The influence of other confounding factors on the olfactory threshold scores was not significant.

### 3.4. Influence of TNF-α Inhibitor Treatment on the Chemosensory Function in IBD

Comparing the olfactory and gustatory function of the IBD patients treated with different therapies, there was no significant difference between the TNF-α inhibitor treatment group (*n* = 50) and the non-TNF-α inhibitor treatment group (*n* = 53). To further investigate the impact of TNF-α inhibitor treatment, we followed 12 patients (eight men, four women, mean age 35.4 ± 11.9 years) who received TNF-α inhibitor treatment for the first time and examined their chemosensory function again after 4 months of treatment. In total, 50% of them were treated with infliximab, 25% with adalimumab, and 25% with golimumab. The clinical characteristics and chemosensory function changes after 4 months of TNF-α inhibitor treatment are shown in Table 6. The clinical disease activity grade and fecal calprotectin of IBD patients, a crucial indicator of intestinal inflammation, significantly decreased after 4 months of anti-TNF-α therapy (*p* = 0.004, *p* < 0.001, respectively). We found that their TDI scores significantly increased after 4 months of TNF-α inhibitor treatment (*p* = 0.038).

## 4. Discussion

In this study, we found that IBD patients were mostly normosmic, but they appeared to be slightly more likely to exhibit decreased olfactory function, as compared to healthy people. Fischer et al. also reported that CD patients showed lower amplitudes of olfactory event-related potentials (OERPs) indicating decreased olfactory function [33]. Similarly, this compares to some degree with data by Steinbach et al., who reported that IBD patients exhibit a minor reduction of olfactory and gustatory function. As in the present study, changes were mainly found for olfactory thresholds, and not for the suprathreshold measurements of odor identification and odor discrimination [34]. This pattern of changes in olfactory function may indicate that changes in olfactory sensitivity are more due to changes at the level of the olfactory mucosa than to central-nervous changes in the processing of olfactory information [35,36].

Patients with IBD often have extra-intestinal inflammatory manifestations, which could be caused by the extension/translocation of immune responses from the intestine [37]. Increased levels of various key inflammatory mediators, including IL-6, TNF-α, and IFN-γ, are found in the serum of IBD patients [38]. Because it was shown that inflammatory mediators are important in the pathogenesis of olfactory dysfunction [39,40,41], it appears possible that systemic inflammation in IBD could result in a progressive loss of olfactory neurons and/or inhibit the regeneration of olfactory neurons. Therefore, we speculate that the slight change in olfactory function could be due to the systemic inflammatory response in IBD patients. Future studies should look directly at signs of inflammation at the olfactory epithelium level, possibly using brush biopsies [42].

Surprisingly, our results showed that moderate–severe patients had a higher olfactory function compared to remission and mild patients. Although immune activation (i.e., enlargement of the immune cells and release of the inflammatory cytokines) in the brain is commonly regarded as a harmful event, it actually could promote neurogenesis when triggering the neuroprotective function of certain immune cells [43]. Furthermore, olfactory stem cells possess a remarkable regenerative capacity and could thus continuously replenish lost olfactory sensory neurons (OSNs) [44]. After extensive olfactory epithelium lesions that depleted the major olfactory sensory neurons proliferating population-globose basal cells (GBCs), horizontal basal cells (HBCs) were induced to proliferate and contribute to the repair of the olfactory epithelium [45,46]. It may be speculated that olfactory sensory neuron regeneration was more active in patients with moderate–severe IBD.

Furthermore, we found that the alteration of olfactory function mainly was manifested for olfactory thresholds, which, to some degree, represent more peripheral olfactory function than central-nervous functions [35]. This may indicate that the lesion is more on a peripheral level, the olfactory mucosa, which in turn may suggest that olfactory dysfunction in IBD patients is due to mucosal inflammation. Unlike the present results, previous research did not reveal an association between disease activity and olfactory function [34]. This may be due to our study having a larger and broader patient population with various disease activity ratings. The difference between the various studies in terms of sample size may account for the distinct results.

In addition, the present study demonstrated that BMI is inversely correlated with olfactory function in patients with IBD, which is in line with previous studies [47]. The olfactory system serves as an internal sensor of the chemical state and energy balance and could influence food choice and modulate body metabolism. The aroma of food was shown to improve the feeling of satiation and modulate food intake [48]. In turn, metabolic disorders could affect olfactory abilities in response to the feeding state and modify the pattern of brain activation for food odors [49]. Therefore, patients with impaired olfactory function tend to take in more food to increase satiety, which results in a gain in body weight.

However, we did not observe a significant taste disorder in IBD patients in the current investigation, which was displayed in the previous studies [34,50]. This discrepancy may arise from different taste testing tools involved in the studies: Melis et al. and Steinbach et al. utilized “taste strips” to assess the taste threshold of IBD patients, whereas our study screened their suprathreshold taste function with “taste sprays”. Future studies to comprehensively assess IBD patients’ taste function are needed.

Our results in a small subgroup (*n* = 12) also suggest that patients exhibited an increased olfactory function after TNF-α inhibitor treatment. TNF-α inhibitors are widely applied in the treatment of IBD, including both CD and UC [51]. The pro-inflammatory cytokine TNF-α was identified as the pivotal factor in the inflammatory cascade causing chronic systemic inflammation in IBD. Therapeutic anti-TNF-α antibodies mitigate this inflammatory process. The influence of TNF-α inhibitor on inflammation-related olfactory loss was investigated using a mouse model in the previous study [52]. The administration of TNF-α inhibitors could result in the interruption of inflammation-induced olfactory loss and facilitate olfactory neuroepithelial regeneration.

In this study, we examined chemosensory functions in a large sample of patients with inflammatory bowel disease and their association with clinical disease activity. However, there are some limitations of the study. First, our study did not include a healthy control group and could not directly delineate the difference between olfactory and gustatory function in patients with IBD and healthy people. Second, we only screened gustatory function using “taste sprays”. The exact gustatory function in IBD patients should be investigated using more refined tools in future studies. Finally, we only followed up with a small number of the patients to study the effect of TNF-α inhibitor treatment on chemosensory function. Our results must be confirmed in a larger sample of patients. In addition, future studies should pay more attention to inflammatory and dietary parameters and should also include healthy individuals.

## 5. Conclusions

In conclusion, IBD patients were more likely to present with hyposmia. Olfactory thresholds were mainly affected. They were significantly associated with clinical disease activity and BMI. As shown in a subgroup, treatment with TNF-α inhibitors appeared to improve olfactory function.

## Figures and Tables

**Table 1:** Demographics and clinical characteristics of IBD patients.

	Remission (*n* = 38)	Mild (*n* = 45)	Moderate–Severe (*n* = 20)	Total (*n* = 103)	*p*-Value
Age, years	42.0 ± 12.5	40.3 ± 12.5	37.3 ± 11.0	40.3 ± 1.2	0.39
Gender, women	19 (50.0%)	25 (55.6%)	7 (35.0%)	51 (49.5%)	0.31
BMI, kg/m^2^	23.96 ± 3.96	24.59 ± 5.28	24.56 ± 4.39	24.35 ± 0.46	0.81
Type of disease					0.86
UC	15 (39.5%)	17 (37.8%)	9 (45.0%)	41 (39.8%)	
CD	23 (60.5%)	28 (62.2%)	11 (55.0%)	62 (60.2%)	
Duration of disease, years	18.4 ± 9.9	12.9 ± 7.9 ^a^	9.7 ± 7.8 ^a^	14.3 ± 0.9	<0.001 **
CRP, mg/L	2.38 ± 3.43	4.76 ± 6.90	6.97 ± 14.31	4.30 ± 0.80	0.11
Blood leucocytes count, GPt/L	6.69 ± 1.94	7.08 ± 2.05	8.10 ± 2.56	7.14 ± 0.21	0.06
Fecal Calprotectin, µg/g	39 ± 30	1017 ± 1617	1538 ± 3324	870 ± 231	0.06
ADS-L depression score	15.71 ± 4.72	15.09 ± 4.43	12.05 ± 5.31 ^a, b^	14.73 ± 0.48	0.018 *
TNF-inhibitor use, *n*	16 (42.1%)	27 (60.0%)	7 (35.0%)	50 (48.5%)	0.10

Results are expressed as mean ± SD or *n* (%). ^a^ Significantly different with respect to remission IBD. ^b^ Significantly different with respect to mild IBD. * *p* < 0.05; ** *p* < 0.01.

**Table 2:** Olfactory function of IBD patients for different age groups [*n* (%)]. None of the participants was functionally anosmic.

Age Group (Years)	IBD Patients		Normative Data *	
	Hyposmia, *n* (%)	Normosmia, *n* (%)	Hyposmia, %	Normosmia, %
11–20	1 (50)	1 (50)	19.5	77.1
21–30	5 (24)	16 (76)	9.6	79.4
31–40	11 (31)	25 (69)	10.7	83.5
41–50	4 (19)	17 (81)	20.7	75.2
51–60	8 (42)	11 (57)	28.8	68.1
61–70	2 (100)	0 (0)	38.5	59.4
71–80	0 (0)	2 (100)	60.0	36.5
Total	31 (30)	72 (70)		

* Oleszkiewicz, A. et al. [29].

**Table 3:** Chemosensory function differences between patients with remission, mild, and moderate–severe IBD.

	Remission (*n* = 38)	Mild (*n* = 45)	Moderate–Severe (*n* = 20)	*p*-Value
Threshold scores	6.58 ± 2.75	5.88 ± 2.52	8.64 ± 1.97 ^a,b^	<0.001 **
Discrimination scores	12.68 ± 1.40	12.76 ± 2.16	12.75 ± 1.74	0.98
Identification scores	13.47 ± 1.47	13.67 ± 1.45	13.60 ± 1.31	0.83
TDI scores	32.74 ± 3.91	32.30 ± 4.80	34.99 ± 3.35	0.06
Taste sprays scores	3.97 ± 0.16	3.93 ± 0.25	3.90 ± 0.31	0.50

^a^ Significantly different with respect to remission IBD. ^b^ Significantly different with respect to mild IBD. ** *p* < 0.01.

**Table 4:** Associations between olfactory and gustatory tests and selected clinical and laboratory variables in IBD patients.

	Threshold Scores	Discrimination Scores	Identification Scores	TDI Scores	Taste Sprays Scores
Age, years					
r	−0.17	−0.02	−0.14	−0.16	0.07
*p*	0.09	0.83	0.16	0.11	0.49
BMI					
r	−0.25	−0.08	0.08	−0.17	−0.15
*p*	0.010 **	0.41	0.45	0.09	0.13
Duration of disease					
r	0.02	0.03	−0.06	0.00	0.18
*p*	0.85	0.81	0.58	0.97	0.08
CRP					
r	−0.08	0.02	−0.01	−0.05	−0.01
*p*	0.42	0.83	0.91	0.65	0.92
Blood leucocytes count					
r	0.03	−0.06	−0.07	−0.03	−0.16
*p*	0.74	0.53	0.48	0.77	0.12
Fecal calprotectin					
r	0.12	−0.16	0.00	0.00	0.04
*p*	0.30	0.17	0.98	0.99	0.72
ADS-L depression score					
r	−0.02	0.13	0.10	0.08	0.12
*p*	0.86	0.19	0.32	0.44	0.22

** *p* < 0.01.

**Table 5:** Multiple linear regression analysis to evaluate the contribution of clinical parameters on olfactory threshold scores.

	Unstandardized Coefficients		Standardized Coefficients		
	B	SE	β	t	*p*
BMI	−0.16	0.06	−0.27	−0.28	0.006 **
Clinical disease activity grade	0.84	0.35	0.23	2.41	0.018 *
Model	R	R^2^	adjusted R^2^		
	0.34	0.12	0.10		

* *p* < 0.05; ** *p* < 0.01.

**Table 6:** Clinical characteristics and chemosensory function change after 4 months of TNF-α inhibitor treatment (*n* = 12).

	Before Treatment	After Treatment	*p*-Value
Age, years	35.4 ± 11.9	-	-
Gender, women	4 (33.3%)	-	-
Duration of disease, years	7.3 ± 2.1	-	-
Clinical disease activity grade			0.004 **
Remission	0	3 (25%)	
Mild	3 (25%)	7 (58.3%)	
Moderate–Severe	9 (75%)	2 (16.7%)	
BMI, kg/m^2^	24.53 ± 5.24	24.77 ± 5.07	<0.001 **
CRP, mg/L	4.66 ± 4.80	2.89 ± 4.53	0.49
Blood leucocytes count, GPt/L	8.81 ± 2.25	6.88 ± 2.24	0.41
Fecal Calprotectin, µg/g	2297 ± 4173	1339 ± 3127	<0.001 **
ADS-L depression score	12.6 ± 6.2	16.1 ± 4.9	0.020 *
TNF-α inhibitor treatment, *n* (%)			
Infliximab	-	6 (50%)	
Adalimumab	-	3 (25%)	
Golimumab	-	3 (25%)	
Chemosensory function			
Threshold scores	7.65 ± 1.98	8.31 ± 1.92	0.42
Discrimination scores	12.83 ± 1.64	13.75 ± 1.36	0.09
Identification scores	13.75 ± 1.22	14.25 ± 1.29	0.14
TDI scores	34.23 ± 2.73	36.31 ± 2.94	0.038 *
Taste sprays scores	3.83 ± 0.39	3.92 ± 0.29	0.59

Results are expressed as mean ± SD or n (%). * *p* < 0.05; ** *p* < 0.01.

## Data Availability

No new data were created or analyzed in this study. Data sharing is not applicable to this article.
